# *CmDUF239-1* Improves the Salt Tolerance of Grafted Melon by Enhancing Antioxidant Capacity and Na^+^/K^+^ Homeostasis

**DOI:** 10.3390/plants14172670

**Published:** 2025-08-27

**Authors:** Yanjun Liu, Zhanming Tan, Lulu Meng, Yang Li, Yuquan Peng

**Affiliations:** 1 Hubei Key Laboratory of Spices & Horticultural Plant Germplasm Innovation & Utilization, College of Horticulture and Gardening, Yangtze University, Jingzhou 434023, China; liuyanjun_02@163.com (Y.L.); lulu_meng1997@163.com (L.M.); 18062385315@163.com (Y.L.); 2 Xinjiang Production & Construction Corps Key Laboratory of Facility Agriculture, College of Horticulture and Forestry Sciences, Tarim University, Alar 843300, China; tlmdxzkytzm@taru.edu.cn

**Keywords:** *DUF* gene, grafting, salt tolerance, antioxidant enzyme activity, Na^+^/K^+^ transport, transcriptome analysis

## Abstract

Salt stress poses a substantial challenge to melon cultivation, but grafting techniques have shown promise in enhancing salt tolerance. This study aims to identify key genes involved in salt tolerance within melon rootstocks. The salt tolerance of four melon cultivars was evaluated, revealing that ‘ST2’ exhibited salt sensitivity, whereas ‘XZM17’ demonstrated salt tolerance. Grafting experiments indicated that salt-sensitive melons benefit significantly from being grafted onto salt-tolerant rootstocks. Transcriptome analysis further identified the *CmDUF239-1* gene as a critical factor contributing to improved salt tolerance in grafted melons. Functional studies demonstrated that knocking out *CmDUF239-1* reduces salt tolerance, reflected in decreased activities of antioxidant enzymes (SOD, POD, CAT) and diminished expression levels of related genes (*CmSOD1*, *CmPRX53-1*, *CmPRX53-2*, *CmCAT2*). Conversely, overexpression of *CmDUF239-1* leads to enhanced enzyme activity and gene expression, along with improved Na^+^/K^+^ homeostasis, evidenced by decreased Na^+^ accumulation and increased K^+^ absorption. Furthermore, *CmDUF239-1* overexpression upregulated Na^+^/K^+^ transport-related genes (*CmSOS1*, *CmNHX6*, *CmKUP3*, *CmSKOR*), whereas *CmDUF239-1* knockout had the opposite effect. These findings indicate that *CmDUF239-1* plays a dual role in promoting salt tolerance by regulating antioxidant defenses and ion transport, contributing to our understanding of the molecular mechanisms behind grafting-induced salt tolerance and providing insights for the breeding of resilient melon varieties.

## 1. Introduction

Soil salinization currently affects approximately 1.125 billion hectares of land globally and is worsening due to inadequate leaching in facility agriculture [1,2]. Under salinity stress, sodium ions (Na^+^) can exert ionic toxic effects on plants, inhibiting growth and, in severe cases, causing root rot and plant wilting [3,4]. To cope with salt stress, plants have evolved two key strategies: regulating the antioxidant system and maintaining Na^+^/K^+^ homeostasis [5,6]. Salt stress induces the accumulation of reactive oxygen species (ROS), which can damage essential biological macromolecules [7,8]. In response, plants activate antioxidant enzyme systems, including superoxide dismutase (SOD) and catalase (CAT), to convert ROS into harmless molecules and protect cellular structures from oxidative damage [9,10,11]. Additionally, maintaining Na^+^/K^+^ balance is essential for various physiological processes, including osmotic regulation and signal transduction [12]. Plants have evolved ion transport proteins, such as *SOS1* and *NHX1* genes, to regulate ion concentrations and enhance salt tolerance [13,14,15,16,17].

The Domain of Unknown Function (DUF) family is widely distributed across species and has been demonstrated to enhance plant salt tolerance by modulating antioxidant capacity and ion homeostasis [18,19,20]. For example, overexpression of OsDUF6 in rice enhances antioxidant enzyme activities and maintains Na^+^/K^+^ homeostasis, thereby improving salt tolerance [21]. Similarly, GmDUF4228-70 in soybean enhances antioxidant enzyme activities and mitigates oxidative damage under salt stress [22]. In *Arabidopsis thaliana*, the *ZmDUF1644* gene from maize improves salt tolerance by regulating intracellular Na^+^/K^+^ homeostasis [23].

Grafting techniques also enhance salt tolerance in vegetable crops by strengthening antioxidant capacity and regulating Na^+^/K^+^ homeostasis [24,25]. For instance, grafting salt-tolerant pumpkin rootstocks onto cucumbers significantly enhances antioxidant enzyme activities and improves salt tolerance [26]. In grafted tomatoes, grafting increases the activity of SOD in the leaves of the scion, contributing to enhanced salt tolerance [27]. Furthermore, grafting influences the absorption and distribution of Na^+^ and K^+^, thereby regulating Na^+^/K^+^ homeostasis [28,29,30,31].

As an economically important crop, melon (*Cucumis melo* L.) faces growth and yield limitations imposed by diverse environmental stresses, among which salt stress is particularly detrimental [32,33,34,35]. Grafting salt-tolerant melon varieties onto salt-sensitive cultivars effectively enhances the latter’s salt tolerance. However, the key salt-responsive genes in rootstocks and the molecular mechanisms by which they regulate salt tolerance in grafted melons remain largely unclear. This study initially screened the salt-sensitive melon variety ‘ST2’ and the salt-tolerant variety ‘XZM17’. Subsequently, grafting was conducted using the salt-tolerant melon as rootstock and the salt-sensitive melon as scion. Our findings demonstrated that grafting significantly improved the salt tolerance of the salt-sensitive melon. Further transcriptome analysis identified the uncharacterized protein gene *CmDUF239-1* in the root system of salt-tolerant melon rootstocks, revealing its crucial role in regulating salt tolerance in grafted melons. Additionally, root transformation experiments indicated that *CmDUF239-1* enhances salt tolerance in grafted melons by upregulating genes involved in antioxidant enzyme synthesis and Na^+^/K^+^ transport. This regulation ultimately strengthens antioxidant capacity and maintains Na^+^/K^+^ homeostasis. This study not only deepens our understanding of the molecular mechanisms by which grafting improves the salt tolerance of vegetables but also provides valuable insights for the molecular breeding of salt tolerance in melons.

## 2. Results

### 2.1. Grafting Enhances the Salt Tolerance of Melons

To assess salt sensitivity and tolerance, three melon cultivars (‘ST2’, ‘LBS9’, and ‘BCG’) and one pure line (‘XZM17’) were subjected to salinity stress using 200 mM NaCl [1]. Compared to normal conditions, salt treatment significantly inhibited the growth of all four melon materials (Figure S1a). Specifically, the shoot dry weights were reduced by 69.8%, 41.5%, 39.5%, and 37.1% for ‘ST2’, ‘LBS9’, ‘BCG’, and ‘XZM17’, respectively (Figure S1b). Similarly, root dry weights decreased significantly by 85.1%, 62.6%, 47.0%, and 41.3%, respectively (Figure S1c). Additionally, both the leaves and root systems exhibited signs of damage. The malondialdehyde (MDA) content in the leaves increased significantly, with values rising by 183.8%, 159.0%, 174.1%, and 93.7% for ‘ST2’, ‘LBS9’, ‘BCG’, and ‘XZM17’, respectively (Figure S1d). In the root systems, MDA levels rose significantly by factors of 8.2, 5.6, 5.7, and 3.7, respectively (Figure S1e). The relative electrical conductivity (REC) of the leaves also showed significant increases, with values rising by 5.4, 2.7, 2.1, and 1.5 times for ‘ST2’, ‘LBS9’, ‘BCG’, and ‘XZM17’, respectively (Figure S1f). REC measurements for the root systems increased by 171.1%, 46.4%, 41.0%, and 0.5%, respectively (Figure S1g). These results indicate that among the four tested melon varieties, ‘ST2’ exhibits the lowest salt tolerance and is classified as a salt-sensitive variety, while ‘XZM17’ demonstrates the highest salt tolerance and is categorized as salt-tolerant.

**Figure 1:**
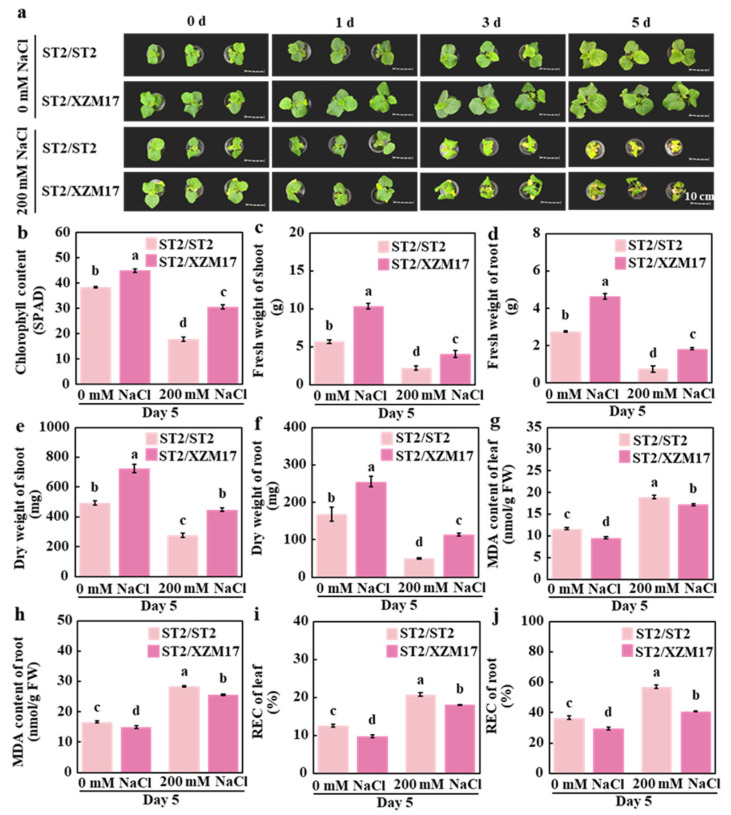
Effects of grafting on salt tolerance in melon seedlings: The phenotypes of self-grafted and grafted melon under a 200 mM NaCl treatment for 0, 1, 3, and 5 days are shown (**a**). Chlorophyll content of self-grafted and grafted melon after 5 days of 200 mM NaCl treatment is presented (**b**), along with their fresh weight of shoot (**c**), fresh weight of root (**d**), dry weight of shoot (**e**), dry weight of root (**f**), MDA content of leaf (**g**), MDA content of root (**h**), REC of leaf (**i**), and REC of root (**j**). Mean ± SE (n = 3). Different lowercase letters indicate significant differences among treatments (*p* < 0.05). ST2/ST2: Self-grafted melon using ‘ST2’ as both the rootstock and scion, ST2/XZM17: Grafted melon with ‘XZM17’ as the rootstock and ‘ST2’ as the scion.

To elucidate the role of grafting in melon salt tolerance, ‘ST2’ (salt-sensitive) and ‘XZM17’ (salt-tolerant) were used as rootstocks, while ‘ST2’ served as the scion. Salinity stress was applied at 200 mM NaCl following the establishment of two graft combinations: ST2/ST2 and ST2/XZM17. The results indicated that under control conditions, after 5 days of treatment, the growth of the ST2/XZM17 combination was superior to that of ST2/ST2 (Figure 1a). Specifically, the leaves of ST2/XZM17 appeared greener, exhibiting higher chlorophyll content, as well as increased fresh and dry weights for both the shoot and root systems. The phenotype of ST2/XZM17 remained superior to that of ST2/ST2 under salt stress conditions (Figure 1a). The SPAD value of the leaves in ST2/XZM17 increased by 70.8% (Figure 1b), while the fresh weight of the shoot and roots rose by 86.3% and 143.4%, respectively (Figure 1c,d). Additionally, the dry weight of these tissues increased by 61.7% and 126.9%, respectively (Figure 1e,f), indicating lower levels of damage. The MDA content in the leaves and roots decreased by 51.4% and 45.1%, respectively (Figure 1g,h), and the REC values in the leaves and roots decreased by 44.1% and 36.1%, respectively (Figure 1i,j). When compared to the control group, under salt stress, the SPAD values of the leaves in ST2/ST2 and ST2/XZM17 decreased by 53.3% and 32.1%, respectively (Figure 1b). The fresh weight of the shoot decreased by 61.3% and 60.5%, while the fresh weight of the root systems decreased by 72.8% and 60.5%, respectively (Figure 1c,d). Furthermore, the dry weight of the shoot decreased by 43.8% and 38.1%, and the dry weight of the root systems decreased by 70.4% and 55.5%, respectively (Figure 1e,f). Notably, the level of damage in ST2/XZM17 was also lower compared to ST2/ST2. In conclusion, grafting with salt-tolerant rootstock significantly enhances the salt tolerance of melon.

### 2.2. CmDUF239-1 in the Rootstock Root System: A Key Gene for Enhancing Salt Tolerance in Grafted Melon

**Figure 2:**
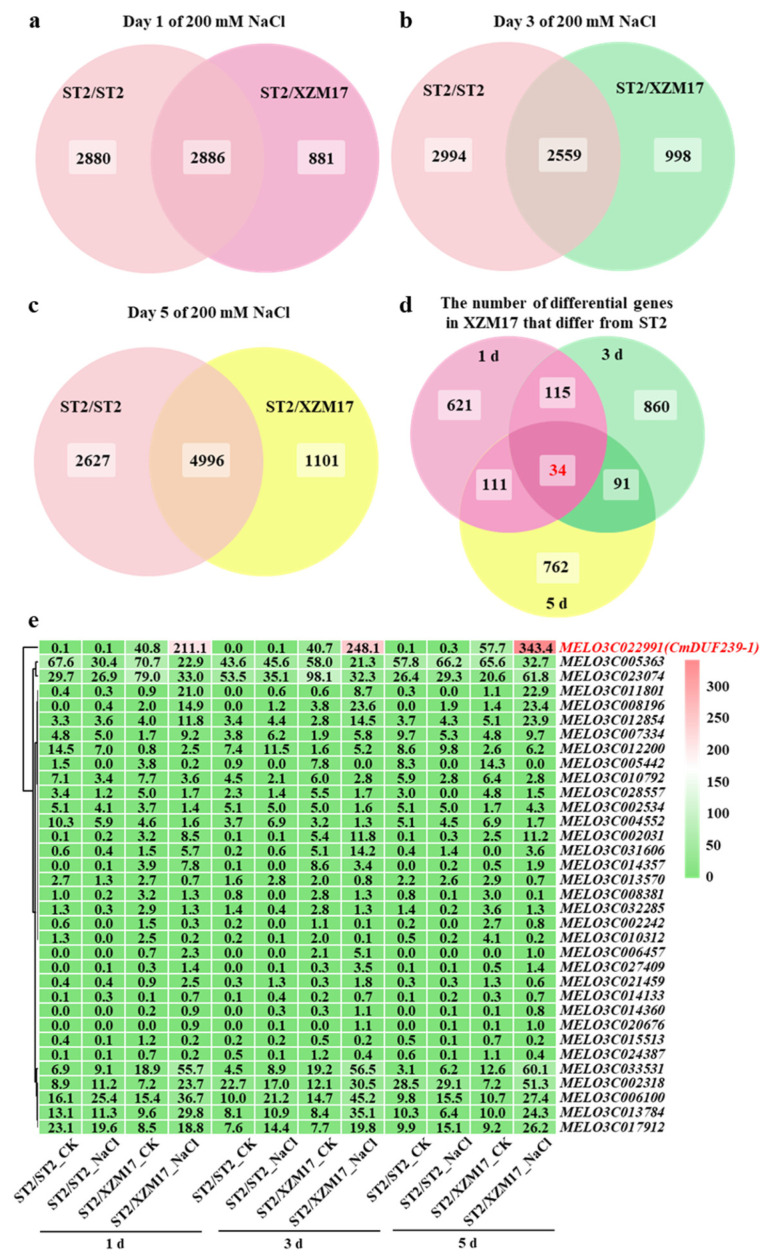
Venn diagram and heatmap analysis of differential genes in the roots of self-grafted and grafted melon under salt stress: Venn diagram analysis of differential genes in the roots of self-grafted and grafted melon treated with 200 mM NaCl for 1 day (**a**), 3 days (**b**), and 5 days (**c**). (**d**) Venn diagram comparing the unique differential genes in the roots of grafted melon versus self-grafted melon after 1, 3, and 5 days of 200 mM NaCl treatment. (**e**) Heatmap analysis of the expression levels of 34 differential genes that respond to salt stress in the roots of grafted melon compared to self-grafted melon at 1, 3, and 5 days following the 200 mM NaCl treatment. ST2/ST2: Self-grafted melon using ‘ST2’ as both the rootstock and scion, ST2/XZM17: Grafted melon with ‘XZM17’ as the rootstock and ‘ST2’ as the scion.

Considering the critical role of the rootstock root system in conferring salt tolerance to grafted melons, this study aims to identify key genes involved in grafting-mediated enhancement of salt tolerance. To achieve this, transcriptome sequencing was conducted on the root systems after exposure to salt treatment for 1, 3, and 5 days. Initially, principal component analysis (PCA) was performed on the transcriptome data. The PCA results indicated that all three samples from each treatment fell within the 90% confidence ellipse, confirming the accuracy and reliability of the transcriptome data (Figure S2a–c). Following this, the number of differentially expressed genes (DEGs) was analyzed. It was observed that during the salt treatment, the ‘ST2’ rootstock exhibited 5766, 5553, and 7623 DEGs at 1, 3, and 5 days, respectively. In contrast, the ‘XZM17’ rootstock showed 3767, 3557, and 6097 DEGs over the same time points (Figure S2d). Further analysis of these differentially expressed genes (DEGs) using Venn diagrams revealed that at 1, 3, and 5 days of salt treatment, 881, 998, and 1101 genes, respectively, responded exclusively to salt stress in the ‘XZM17’ root system, with no corresponding expression detected in the ‘ST2’ root system (Figure 2a–c). By analyzing the intersection of these genes, 34 genes were identified that responded to salt stress at all time points in the ‘XZM17’ root system but showed no response in the ‘ST2’ root system (Figure 2d). A heat map analysis of these 34 genes demonstrated that the expression level of *MELO3C022991* was the highest. Under salt stress, the expression level of this gene in the root system of ‘XZM17’ was significantly greater than that in ‘ST2’ (Figure 2e). Comparative analysis of the protein sequence with that of *Arabidopsis thaliana* classified the gene within the DUF239 family, leading to its designation as *CmDUF239-1*. Quantitative polymerase chain reaction (qPCR) results further indicated that under control conditions, the expression levels of *CmDUF239-1* did not differ significantly between the root systems of ‘XZM17’ and ‘ST2’. However, following 1, 3, and 5 days of salt treatment, the expression level of *CmDUF239-1* in the root system of ‘XZM17’ increased significantly by 3.7, 3.5, and 3.0 times, respectively, compared to ‘ST2’ (Figure S3a–c). In conclusion, *CmDUF239-1* emerges as a key gene that facilitates the enhancement of salt tolerance in grafted melon. The expression levels of the *CmDUF239-1* gene were quantitatively analyzed in various tissues of melon ‘XZM17’ following one day of salt stress. Under control conditions, no statistically significant differences were observed in *CmDUF239-1* expression among roots, stems, and leaves. However, salt stress induced a marked upregulation of *CmDUF239-1* expression in all examined tissues (Figure S3d), suggesting an active role of *CmDUF239-1* in the plant’s salt stress response across multiple organs. Further subcellular localization analysis in tobacco leaves revealed that CmDUF239-1 was discontinuously distributed along the cell membrane, implying a potential mechanism through which the protein enhances local membrane robustness to mitigate salt-induced stress (Figure S3e).

### 2.3. CmDUF239-1 Positively Regulates the Salt Tolerance of Grafted Melon

**Figure 3:**
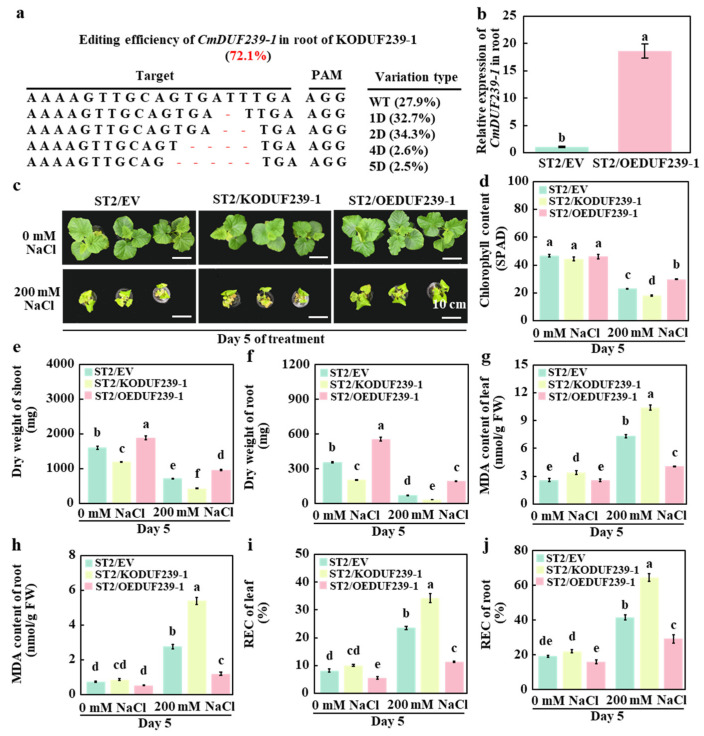
Effects of *CmDUF239-1* gene knockout and overexpression on the salt tolerance of grafted melon: (**a**) Gene editing efficiency of *CmDUF239-1* in the roots of ST2/KODUF239-1 grafted melon. (**b**) Overexpression effects of *CmDUF239-1* in the roots of ST2/OEDUF239-1 grafted melon. Phenotypes (**c**), chlorophyll content (**d**), dry weight of shoot (**e**), dry weight of root (**f**), MDA content in leaves (**g**) and roots (**h**), and relative electrical conductivity (REC) in leaves (**i**) and roots (**j**) of grafted melon with root knockout and overexpression of *CmDUF239-1* gene under salt stress. Mean ± SE (n = 3). Different lowercase letters indicate significant differences between treatments (*p* < 0.05). ST2/EV: Grafted melon transformed with an empty vector in the roots; ST2/KODUF239-1: Grafted melon with *CmDUF239-1* knockout in the roots; ST2/OEDUF239-1: Grafted melon with *CmDUF239-1* overexpression in the roots.

To investigate the role of the *CmDUF239-1* gene in enhancing salt tolerance in grafted melon, various vectors were transformed into the root system of the rootstock. Specifically, we utilized an empty vector, a *CmDUF239-1* gene editing vector, and a *CmDUF239-1* gene overexpression vector. The salt-sensitive cultivar ‘ST2’ served as the scion for grafting. The resulting grafted melon included those with root transformation using an empty vector (ST2/EV), those subjected to *CmDUF239-1* gene editing (ST2/KODUF239-1), and those exhibiting *CmDUF239-1* gene overexpression (ST2/OEDUF239-1). The efficiency of *CmDUF239-1* gene editing in the root system of ST2/KODUF239-1 was assessed using HiTom technology, yielding a result of 72.1% (Figure 3a). Quantitative PCR (qPCR) analysis revealed that CmDUF239-1 expression in the root system of ST2/OEDUF239-1 was increased 17.3-fold relative to ST2/EV. (Figure 3b). Following salt exposure, phenotypic assessment demonstrated that under control conditions, ST2/KODUF239-1 plants displayed reduced size relative to ST2/EV plants, with dry weights of shoots and roots significantly diminished. Conversely, the ST2/OEDUF239-1 group exhibited enhanced growth (Figure 3c). Under salt stress, the phenotype of ST2/KODUF239-1 deteriorated further; leaf SPAD values decreased by 21.6% (Figure 3d), while dry weights of the shoot and root system diminished by 39.6% and 50.9%, respectively (Figure 3e,f). Consequently, these plants experienced greater damage, reflected by a 41.8% increase in MDA content in leaves and a staggering 95.2% increase in roots (Figure 3g,h). Additionally, REC rose by 45.8% in leaves and 55.2% in roots (Figure 3i,j). In contrast, the ST2/OEDUF239-1 phenotype improved under salt treatment. SPAD values increased by 29.6% (Figure 3d), while dry weights of the shoot and root system rose by 35.6% and 171.3%, respectively (Figure 3e,f). These plants showed lower levels of damage, with MDA content in leaves and roots decreasing by 44.5% and 56.6%, respectively (Figure 3g,h). The REC of leaves and roots also decreased by 51.8% and 29.6% (Figure 3i,j). Furthermore, when comparing responses to salt treatment among the groups, the SPAD values of the leaves dropped by 50.9%, 59.4%, and 35.3% for ST2/EV, ST2/KODUF239-1, and ST2/OEDUF239-1, respectively (Figure 3d). The shoot dry weights decreased by 55.7%, 64.1%, and 49.0% (Figure 3e). The dry weight of the root system fell by 79.9%, 82.8%, and 64.9%, respectively (Figure 3f). The MDA content in the leaves was elevated by 1.9-, 2.1-, and 0.6-fold, and in the roots by 2.8-, 5.2-, and 1.3-fold (Figure 3g,h). Additionally, the REC of leaves increased by 1.9-, 2.4-, and 1.1-fold, with root REC rising by 1.2-, 1.9-, and 0.8-fold across the three treatments. (Figure 3i,j). These findings indicate that knocking out *CmDUF239-1* in the root system adversely affects the salt tolerance of grafted melon, while its overexpression enhances this trait, suggesting that *CmDUF239-1* positively regulates salt tolerance in grafted melon.

### 2.4. CmDUF239-1 Enhances Antioxidant Enzyme Activity in Grafted Melon Roots Under Salt Stress

**Figure 4:**
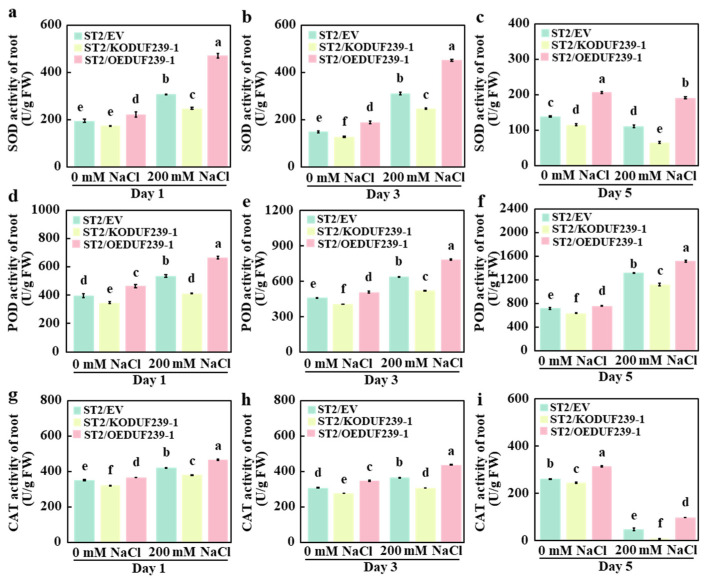
Effects of *CmDUF239-1* knockout and overexpression on antioxidant enzyme activity in the roots of grafted melon: SOD activity in the roots of grafted melon with *CmDUF239-1* knockout and overexpression under 200 mM NaCl treatment for 1 day (**a**), 3 days (**b**), and 5 days (**c**). Peroxidase (POD) activity in the roots of grafted melon under 200 mM NaCl treatment for 1 day (**d**), 3 days (**e**), and 5 days (**f**). CAT activity in the roots of grafted melon under 200 mM NaCl treatment for 1 day (**g**), 3 days (**h**), and 5 days (**i**). Mean ± SE (n = 3). Different lowercase letters indicate significant differences between treatments (*p* < 0.05). ST2/EV: Grafted melon transformed with an empty vector in the roots; ST2/KODUF239-1: Grafted melon with *CmDUF239-1* knockout in the roots; ST2/OEDUF239-1: Grafted melon with *CmDUF239-1* overexpression in the roots.

Enhancing the activity of antioxidant enzymes is a crucial mechanism by which plants resist salt stress. Antioxidant enzyme activities, including SOD, Peroxidase (POD), and CAT, were measured in the roots of grafted melons transformed with an empty vector (ST2/EV), *CmDUF239-1* gene editing vector (ST2/KODUF239-1), and *CmDUF239-1* overexpression vector (ST2/OEDUF239-1) to assess the effect of *CmDUF239-1*. The results indicated that under salt stress conditions, the antioxidant enzyme activity in the ST2/KODUF239-1 root system was significantly reduced compared to ST2/EV. Following 1, 3, and 5 days of treatment, the activities of SOD, POD, and CAT decreased by 19.7%, 20.8%, and 41.2% (Figure 4a–c), 23.4%, 18.4%, and 14.9% (Figure 4d–f), and 9.5%, 16.0%, and 82.1%, respectively (Figure 4g,h). Conversely, the antioxidant enzyme activity in the ST2/OEDUF239-1 root system showed significant increases. After the same treatment durations, SOD activity increased by 52.9%, 45.6%, and 72.7%, respectively (Figure 4a–c); POD activity rose by 24.0%, 22.8%, and 14.9% (Figure 4d–f); and CAT activity enhanced by 10.6%, 20.0%, and 103.1%, respectively (Figure 4g,h). Compared to the control group, the antioxidant enzyme activities in the roots of all three grafted melon types increased during the early stages of salt treatment. However, as treatment time extended, the activities of some enzymes diminished significantly. Notably, SOD activity showed an increase of 57.7%, 42.5%, and 112.1% after 1 day, 108.6%, 92.9%, and 139.7% after 3 days, but then decreased significantly by day 5 (Figure 4a–c). After 1 day, POD activity rose by 35.0%, 18.4%, and 43.5%, respectively. After 3 days, increases of 39.0%, 28.4%, and 54.6% were observed, while at 5 days, POD activity demonstrated variable responses with increases of 84.1%, 76.4%, and 100.2% (Figure 4d–f). CAT activity also exhibited an initial rise, increasing by 19.8%, 18.7%, and 27.2% after 1 day, followed by increases of 18.5%, 10.7%, and 25.9% after 3 days, but decreased significantly by day 5 (Figure 4g,h). These findings suggest that knockout of the *CmDUF239-1* gene significantly reduces the activities of SOD, POD, and CAT in the root systems of grafted melon under salt stress. In contrast, *CmDUF239-1* overexpression markedly enhances the activities of these antioxidant enzymes, indicating that *CmDUF239-1* plays a vital role in improving salt tolerance of grafted melon through upregulation of antioxidant enzyme activities.

### 2.5. CmDUF239-1 Increased the Expression of Antioxidant Oxidase-Related Genes in Grafted Melon Roots Under Salt Stress

**Figure 5:**
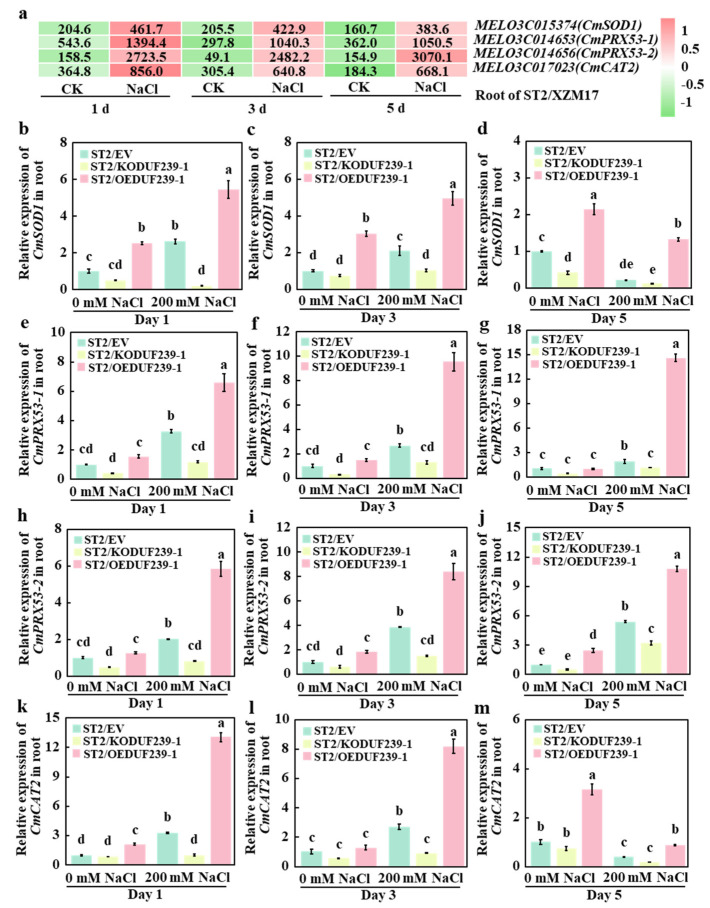
Effects of *CmDUF239-1* knockout and overexpression on the expression of antioxidant enzyme-related genes in the roots of grafted melon: (**a**) Heatmap of antioxidant enzyme-related gene expression in the roots of grafted melon treated with 200 mM NaCl for 1 day, 3 days, and 5 days. Expression levels of the *CmSOD1* gene in the roots of grafted melon with *CmDUF239-1* knockout and overexpression under 200 mM NaCl treatment for 1 day (**b**), 3 days (**c**), and 5 days (**d**). Expression levels of the *CmPRX53-1* gene in the roots of grafted melon under 200 mM NaCl treatment for 1 day (**e**), 3 days (**f**), and 5 days (**g**). Expression levels of the *CmPRX53-2* gene in the roots of grafted melon under 200 mM NaCl treatment for 1 day (**h**), 3 days (**i**), and 5 days (**j**). Expression levels of the *CmCAT2* gene in the roots of grafted melon under 200 mM NaCl treatment for 1 day (**k**), 3 days (**l**), and 5 days (**m**). Mean ± SE (n = 3). Different lowercase letters indicate significant differences between treatments (*p* < 0.05). ST2/EV: Grafted melon transformed with an empty vector in the roots; ST2/KODUF239-1: Grafted melon with *CmDUF239-1* knockout in the roots; ST2/OEDUF239-1: Grafted melon with *CmDUF239-1* overexpression in the roots.

To further investigate the impact of *CmDUF239-1* on the expression levels of root antioxidant oxidase-related genes, the expression of key genes involved in the synthesis of SOD, POD, and CAT was analyzed in the roots of melon rootstocks under salt stress, based on previously obtained transcriptome data. The results indicated that on days 1, 3, and 5 of salt treatment, only four genes—*CmSOD1* (a key gene for SOD synthesis), *CmPRX53-1* and *CmPRX53-2* (key genes for POD synthesis), and *CmCAT2* (a key gene for CAT synthesis)—were significantly upregulated in response to salt stress (Figure 5a). To validate these findings, qPCR technology was employed to analyze the expression levels of *CmSOD1*, *CmPRX53-1*, *CmPRX53-2*, and *CmCAT2* in the roots of three types of grafted melon: ST2/EV (with an empty vector), ST2/KODUF239-1 (with *CmDUF239-1* gene knockout), and ST2/OEDUF239-1 (with *CmDUF239-1* gene overexpression). Under salt treatment, the expression levels of antioxidant oxidase-related genes were significantly decreased in the roots of ST2/KODUF239-1 compared to ST2/EV. Specifically, CmSOD1 expression declined by 92.1%, 50.7%, and 43.9% at 1, 3, and 5 days of treatment, respectively (Figure 5b–d). Furthermore, the *CmPRX53-1* gene expression levels decreased by 63.7%, 51.1%, and 40.3% (Figure 5e–g), while those of the *CmPRX53-2* gene fell by 59.7%, 59.7%, and 60.6% (Figure 5h–j). The expression levels of the *CmCAT2* gene also declined significantly by 69.5%, 69.5%, and 65.9% (Figure 5k–m). In contrast, the expression levels of antioxidant oxidase-related genes in the roots of ST2/OEDUF239-1 were significantly elevated. On days 1, 3, and 5 of treatment, the expression levels of the *CmSOD1* gene increased by 1.1, 1.4, and 5.1 times, respectively (Figure 5b–d). Likewise, the *CmPRX53-1* gene expression levels rose by 1.0, 2.5, and 6.8 times (Figure 5e–g), and the *CmPRX53-2* gene levels increased by 1.9, 1.9, and 1.2 times (Figure 5h–j). The expression levels of the *CmCAT2* gene also saw increases of 3.0, 3.0, and 2.0 times, respectively (Figure 5k–m). When compared to the control group, the expression of antioxidant oxidase-related genes in the roots of ST2/EV, ST2/KODUF239-1, and ST2/OEDUF239-1 showed an initial upregulation during early salt treatment. However, some gene expression levels significantly decreased in the later stages of treatment. These findings indicate that the knockout of *CmDUF239-1* considerably reduced the expression levels of *CmSOD1*, *CmPRX53-1*, *CmPRX53-2*, and *CmCAT2* in the root systems of grafted melon under salt stress, whereas the overexpression of *CmDUF239-1* significantly enhanced the expression levels of these genes. This pattern aligns with changes observed in the activities of SOD, POD, and CAT. It can therefore be concluded that *CmDUF239-1* enhances antioxidant enzyme activity by upregulating the expression of related genes, thereby improving the antioxidant capacity of grafted melon under salt stress.

### 2.6. CmDUF239-1 Promotes Na^+^/K^+^ Homeostasis in Grafted Melon Roots Under Salt Stress

**Figure 6:**
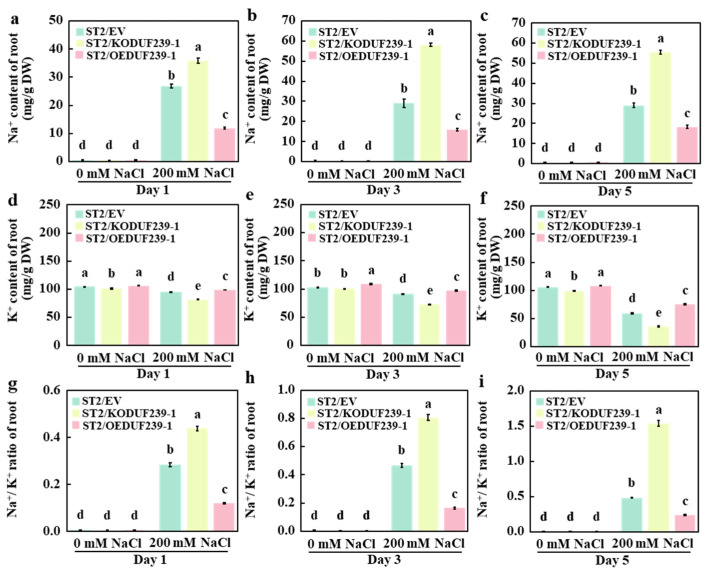
Effects of *CmDUF239-1* knockout and overexpression on the Na^+^/K^+^ homeostasis in the roots of grafted melon: Na^+^ content in the roots of grafted melon with *CmDUF239-1* knockout and overexpression under 200 mM NaCl treatment for 1 day (**a**), 3 days (**b**), and 5 days (**c**). K^+^ content in the roots of grafted melon under 200 mM NaCl treatment for 1 day (**d**), 3 days (**e**), and 5 days (**f**). Na^+^/K^+^ ratio in the roots of grafted melon under 200 mM NaCl treatment for 1 day (**g**), 3 days (**h**), and 5 days (**i**). Mean ± SE (n = 3). Different lowercase letters indicate significant differences between treatments (*p* < 0.05). ST2/EV: Grafted melon transformed with an empty vector in the roots; ST2/KODUF239-1: Grafted melon with *CmDUF239-1* knockout in the roots; ST2/OEDUF239-1: Grafted melon with *CmDUF239-1* overexpression in the roots.

Maintaining sodium–potassium homeostasis is a crucial physiological mechanism that enables plants to cope with salt stress. To further investigate the effects of the *CmDUF239-1* gene on sodium (Na^+^) and potassium (K^+^) homeostasis in grafted melon roots, the concentrations of Na^+^ and K^+^ were measured in roots transformed with an empty vector (ST2/EV), subjected to *CmDUF239-1* gene editing (ST2/KODUF239-1), and overexpressing *CmDUF239-1* (ST2/OEDUF239-1). The results indicated that under salt stress conditions, the ST2/KODUF239-1 root system exhibited a significant increase in Na^+^ content and Na^+^/K^+^ ratio compared to ST2/EV, while K^+^ content significantly decreased. Specifically, after 1, 3, and 5 days of treatment, Na^+^ content increased by 33.2%, 100.0%, and 91.3%, respectively (Figure 6a–c), K^+^ content decreased by 14.1%, 20.5%, and 39.4%, respectively (Figure 6d–f), and the Na^+^/K^+^ ratio increased by 54.8%, 72.7%, and 216.6%, respectively (Figure 6g–i). In contrast, the ST2/OEDUF239-1 root system showed a significant reduction in Na^+^ content and a notable increase in K^+^ content, resulting in a substantial decrease in the Na^+^/K^+^ ratio. On the first, third, and fifth days of treatment, Na^+^ content decreased by 55.8%, 45.2%, and 36.8%, respectively (Figure 6a–c), K^+^ content increased by 4.0%, 7.0%, and 26.4%, respectively (Figure 6d–f), and the Na^+^/K^+^ ratio decreased by 57.5%, 64.8%, and 50.0%, respectively (Figure 6g–i). Furthermore, under salt stress conditions, all three grafted melon types demonstrated a significant increase in Na^+^ content and Na^+^/K^+^ ratio compared to the control group, while K^+^ content significantly decreased. These findings indicate that the knockout of *CmDUF239-1* significantly increases Na^+^ accumulation and decreases K^+^ uptake in the roots of grafted melon, thereby disrupting Na^+^/K^+^ homeostasis. Conversely, overexpression of *CmDUF239-1* effectively reduces Na^+^ content, promotes K^+^ uptake, and maintains Na^+^/K^+^ homeostasis, suggesting that *CmDUF239-1* plays a vital role in improving salt tolerance of grafted melon by regulating Na^+^/K^+^ balance.

### 2.7. CmDUF239-1 Promoted the Expression of Sodium-Potassium Transportation-Related Genes in Grafted Melon Roots Under Salt Stress

**Figure 7:**
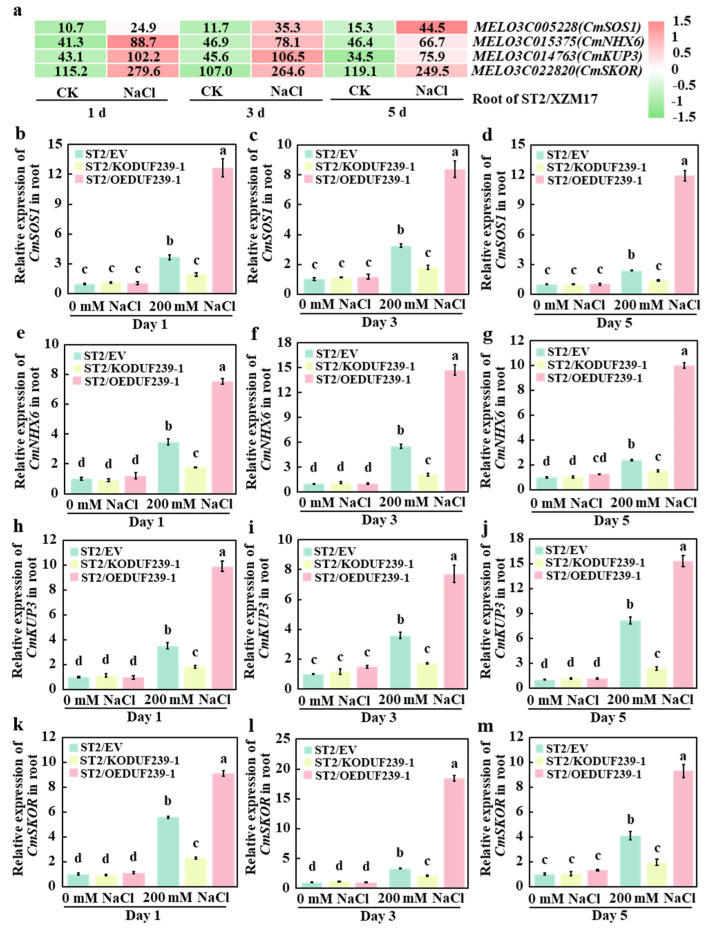
Effects of *CmDUF239-1* knockout and overexpression on the expression of Na^+^/K^+^ transport-related genes in the roots of grafted melon: (**a**) Heatmap analysis of Na^+^/K^+^ transport-related gene expression in the roots of grafted melon treated with 200 mM NaCl for 1 day, 3 days, and 5 days. Expression levels of the *CmSOD1* gene in the roots of grafted melon with *CmDUF239-1* knockout and overexpression under 200 mM NaCl treatment for 1 day (**b**), 3 days (**c**), and 5 days (**d**). Expression levels of the *CmNHX6* gene in the roots of grafted melon under 200 mM NaCl treatment for 1 day (**e**), 3 days (**f**), and 5 days (**g**). Expression levels of the *CmKUP3* gene in the roots of grafted melon under 200 mM NaCl treatment for 1 day (**h**), 3 days (**i**), and 5 days (**j**). Expression levels of the *CmSKOR* gene in the roots of grafted melon under 200 mM NaCl treatment for 1 day (**k**), 3 days (**l**), and 5 days (**m**). Mean ± SE (n = 3). Different lowercase letters indicate significant differences between treatments (*p* < 0.05). ST2/EV: Grafted melon transformed with an empty vector in the roots; ST2/KODUF239-1: Grafted melon with *CmDUF239-1* knockout in the roots; ST2/OEDUF239-1: Grafted melon with *CmDUF239-1* overexpression in the roots.

To further investigate the effect of *CmDUF239-1* on the expression of Na^+^ and K^+^ transport-related genes in the root system, existing transcriptome data were analyzed to assess gene responses to salt stress in melon rootstocks. The results revealed that on days 1, 3, and 5 of salt treatment, only four genes—*CmSOS1* and *CmNHX6* (associated with Na^+^ transport), and *CmKUP3* and *CmSKOR* (involved in K^+^ transport)—were significantly upregulated under salt stress (Figure 7a). Subsequently, qPCR was employed to evaluate the expression levels of these genes in three types of grafted melon: ST2/EV (control group with an empty vector), ST2/KODUF239-1 (*CmDUF239-1* gene-edited), and ST2/OEDUF239-1 (*CmDUF239-1* gene overexpressed). Under salt treatment conditions, the expression levels of Na^+^ and K^+^ transport-related genes in the roots of ST2/KODUF239-1 were significantly lower than those in ST2/EV. Specifically, the expression levels of the *CmSOS1* gene decreased by 47.8%, 44.7%, and 42.9% on days 1, 3, and 5, respectively (Figure 7b–d). Similarly, the expression levels of the *CmNHX6* gene decreased by 49.0%, 61.9%, and 36.0% (Figure 7e–g); the *CmKUP3* gene decreased by 48.6%, 51.4%, and 70.8% (Figure 7h–j); and the *CmSKOR* gene decreased by 58.8%, 36.7%, and 52.5% across the same time points (Figure 7k–m). Conversely, the expression levels of Na^+^ and K^+^ transport-related genes in the roots of ST2/OEDUF239-1 increased significantly. On days 1, 3, and 5 of treatment, the expression levels of the *CmSOS1* gene increased by 2.4, 1.6, and 4.0 times, respectively (Figure 7b–d). The *CmNHX6* gene expression levels rose by 1.2, 1.7, and 3.2 times (Figure 7e–g); the *CmKUP3* gene levels increased by 1.8, 1.2, and 0.9 times (Figure 7h–j); while the *CmSKOR* gene expression levels increased by 0.6, 4.5, and 1.3 times (Figure 7k–m). Additionally, when compared to the control group, salt treatment significantly enhanced the expression levels of Na^+^ and K^+^ transport-related genes in the root systems of ST2/EV, ST2/KODUF239-1, and ST2/OEDUF239-1. In particular, the expression levels of the *CmSOS1*, *CmNHX6*, *CmKUP3*, and *CmSKOR* genes showed significant increases at days 1, 3, and 5 of salt treatment (Figure 7b–m). These findings indicate that under salt stress, *CmDUF239-1* knockout significantly decreases the expression of these transport-related genes, whereas *CmDUF239-1* overexpression markedly upregulates their expression. This expression pattern corresponds with observed changes in Na^+^ and K^+^ contents, suggesting that *CmDUF239-1* plays a critical role in maintaining Na^+^/K^+^ homeostasis in grafted melon by regulating these genes, thereby reducing Na^+^ uptake and enhancing K^+^ absorption.

## 3. Discussion

### 3.1. CmDUF239-1 Positively Regulates Salt Tolerance in Grafted Melon

The DUF gene family has shown considerable promise in enhancing salt tolerance across various crop species. For instance, rice lines overexpressing OsDUF6 exhibited enhanced growth under salt stress, with reductions in root, stem, and leaf lengths significantly less pronounced than those observed in wild-type (WT) plants. This indicates that *OsDUF6* effectively mitigates the inhibitory effects of salt stress on plant development [21]. Similarly, the overexpression of *OsDUF868.12* resulted in markedly enhanced salt tolerance when subjected to 150 mM NaCl treatment, evidenced by increased plant height and overall growth quality. Under more extreme conditions, such as 200 mM NaCl treatment, the survival rate of the *OsDUF868.12* overexpression lines was significantly greater than that of WT plants, while knockout lines exhibited a notable decrease in survival rates. Conversely, the growth of the *OsDUF868.12* knockout line was markedly inhibited under salt stress, exhibiting reduced plant height and severe leaf yellowing [36]. In soybeans, the genes *GmDUF668-8*, *GmDUF668-20*, and *GmDUF668-30* were significantly upregulated in response to salt stress, suggesting their involvement in the soybean’s adaptive mechanisms against salinity [37]. Furthermore, the overexpression of the *GmDUF4228-70* gene substantially improved the growth conditions of soybean plants under salt stress, as these plants displayed higher relative water content (RWC), increased chlorophyll levels, and reduced leaf curling and wilting [22]. In wheat, the *TaDUF966-9B* gene is crucial for salt tolerance; silencing this gene through virus-induced gene silencing (VIGS) technology led to severe leaf curling under salt stress and a marked decline in salt tolerance [38]. *Arabidopsis thaliana* plants expressing the maize ZmDUF1644 gene showed enhanced tolerance to salt stress, as evidenced by significantly higher maximum root lengths and fresh weights relative to WT plants [23]. Overall, these findings highlight the essential role of *DUF* genes in regulating salt tolerance mechanisms across different crops, including the positive regulatory effect of *CmDUF239-1* on salt tolerance in grafted melon.

Through gene editing and overexpression analyses, this research elucidated the role of *CmDUF239-1* in salt tolerance of grafted melon. The results align with established data on *DUF* genes in various crops but present distinct variations. Specifically, the knockout of the *CmDUF239-1* gene resulted in a significant reduction in the salt tolerance of grafted melon, which was evidenced by hindered plant growth (Figure 3c), decreased SPAD values in leaves (Figure 3d), reduced dry weight (Figure 3e,f), and significantly elevated levels of MDA and relative electrical conductivity (REC) (Figure 3g–j). In contrast, overexpression of *CmDUF239-1* markedly enhanced salt tolerance, as indicated by improved plant growth (Figure 3c), increased SPAD values (Figure 3d), higher dry weight (Figure 3e,f), and significant decreases in MDA and REC (Figure 3g–j). These results are consistent with previous studies on *OsDUF6* and *OsDUF868.12* in rice [21], the *GmDUF668* gene family in soybean [37], and *TaDUF966-9B* in wheat [38], collectively demonstrating that the *DUF* gene family positively regulates salt tolerance in diverse crop species. However, it is noteworthy that the editing efficiency of the *CmDUF239-1* gene in this study was only 72.1% (Figure 3a), which may limit the extent to which gene knockout can enhance salt tolerance. This contrasts with observations in wheat, where complete silencing of the *TaDUF966-9B* gene through virus-induced gene silencing (VIGS) technology resulted in a pronounced decrease in salt tolerance [38]. From a methodological perspective, the combination of grafting techniques and gene transformation methods employed in this study offers a novel approach for investigating the functions of root system genes. This differs from traditional methods that perform gene modifications directly on whole plants. The application of this technology allows for a more precise examination of how root genes influence the salt tolerance of grafted plants; however, it may also be constrained by factors such as grafting affinity and gene transformation efficiency. In summary, although the results of this study corroborate previous findings on the DUF gene family’s role in regulating salt tolerance, discrepancies in gene editing efficiency and experimental methodologies were noted. These variations may arise from differences in gene characteristics, crop species, and experimental approaches.

### 3.2. The Role of CmDUF239-1 in Enhancing Salt Tolerance of Grafted Melon by Upregulating Antioxidant Genes

Under salt stress conditions, plant cells accumulate a substantial amount of ROS, such as superoxide anions and hydrogen peroxide [39]. These ROS can damage cell membranes, proteins, and nucleic acids, ultimately inhibiting normal plant growth [6]. Previous studies have indicated that genes from the *DUF* family, along with grafting techniques, can enhance the salt tolerance of crops by regulating antioxidant enzyme activity and gene expression. In rice, overexpression of the *OsDUF6* gene significantly increased the activities of SOD and CAT in roots, stems, and leaves following a 6-day treatment with 200 mM NaCl. The augmented SOD activity catalyzes the conversion of superoxide anions into oxygen and hydrogen peroxide, while enhanced CAT activity facilitates hydrogen peroxide degradation, together mitigating ROS-induced damage and boosting the antioxidant capacity and salt tolerance of rice [21]. Similarly, *OsDUF868.12* was shown to regulate SOD and peroxidase (POD) activities under 200 mM NaCl stress, with transgenic lines exhibiting significantly higher POD activity than wild-type plants, indicating improved scavenging capabilities for hydrogen peroxide and consequently enhanced salt tolerance [36]. In soybeans, plants overexpressing *GmDUF4228-70* demonstrated significantly elevated SOD and CAT activities in their leaves after a 3-day exposure to 200 mM NaCl, highlighting the role of these antioxidant enzymes in promoting salt tolerance [22]. Grafting techniques have been demonstrated to enhance salt tolerance in various crops, including vegetables, primarily by increasing antioxidant enzyme activities. For instance, in grafted cucumbers, significant changes in SOD and CAT activities were observed in both the roots and leaves after pumpkin roots were treated with 100 mM NaCl for 10 days, and the grafted cucumbers received 75 mM NaCl for 7 days. Notably, C/OECDPK20 (cucumbers grafted onto pumpkin roots overexpressing the *CmoCDPK20* gene) exhibited significantly enhanced SOD and CAT activities, alongside a marked upregulation of the *CmoPOD2* gene, suggesting that *CmoCDPK20* improves the antioxidant capacity of grafted cucumbers through modulation of antioxidant enzyme activity and related gene expression [40]. In grafted tomatoes, a 30-day treatment with salt at 8 dS m^−1^ resulted in an 88.87% increase in leaf SOD activity and an 83.87% increase in CAT activity across all grafted combinations compared to control conditions, further supporting the hypothesis that grafting enhances salt tolerance by elevating antioxidant enzyme activity [41]. In grafted chrysanthemums, after 8 days of treatment with 120 mM NaCl, heterografted (HG) plants exhibited significantly higher CAT activity in their roots than both self-rooted (SR) and self-grafted (SG) plants. Following salt treatment, SG plants displayed the highest leaf POD and CAT activities, while SR plants had reduced enzyme levels compared to controls [42]. This supports the hypothesis that cross-grafting improves salt tolerance by enhancing ROS scavenging through increased CAT activity.

This study thoroughly investigated the effects of the *CmDUF239-1* gene on antioxidant enzyme activity and the expression of related genes in the root systems of grafted melon, examining both the functionality of the DUF gene and the influence of grafting techniques. The findings revealed both similarities and notable differences when compared to other crops. Results demonstrated that knockout of the *CmDUF239-1* gene significantly decreased SOD, POD, and CAT activities in roots of grafted melon, indicating an essential role in antioxidant enzyme regulation (Figure 4a–h). Conversely, the overexpression of *CmDUF239-1* resulted in a marked increase in these antioxidant enzyme activities (Figure 4a–h). This aligns with previous research on *OsDUF6* and *OsDUF868.12* in rice [21,36], as well as *GmDUF4228-70* in soybeans [22], all indicating that DUF family genes enhance plant salt tolerance by regulating antioxidant enzyme activity. After 5 days of salt stress, CAT activity in the ST2/KODUF239-1 root system was reduced by 82.1%, contrasting with a 103.1% increase in the ST2/OEDUF239-1 root system, highlighting the pronounced effect of *CmDUF239-1* on CAT activity (Figure 4i). Such a substantial change has not been reported in studies of other crops, possibly reflecting a unique response mechanism of melon roots to salt stress. In addition, this study conducted an in-depth analysis of the expression changes in antioxidant oxidase-related genes. The knockout of the *CmDUF239-1* gene significantly reduced the expression levels of *CmSOD1*, *CmPRX53-1*, *CmPRX53-2*, and *CmCAT2*, whereas the overexpression of *CmDUF239-1* resulted in their significant upregulation (Figure 5b–m). This correlation closely mirrors the changes observed in antioxidant enzyme activities, thereby revealing deeper insights into the molecular mechanisms through which the *DUF* gene enhances the antioxidant capacity of plants by modulating the expression of antioxidant oxidase-related genes. In grafted melon, antioxidant enzyme activities in roots initially increased during the early stages of salt treatment. However, prolonged exposure resulted in a significant decline in the activities of certain enzymes, a pattern that contrasts with observations reported in other grafted crops (Figure 4a–i). SOD and CAT activities in pumpkin roots were significantly enhanced after ten days of salt treatment in grafted cucumbers, whereas grafted tomatoes demonstrated considerable increases in leaf SOD and CAT activities after thirty days of salt exposure [40]. In contrast, a decline in antioxidant enzyme activity was observed in grafted melon roots during the later stages of salt treatment. This reduction may result from inhibited energy metabolism and antioxidant systems in melon roots under prolonged salt stress, leading to decreased enzyme activity. Furthermore, in grafted vegetables, the increased antioxidant enzyme activity observed in cucumbers grafted onto pumpkin roots and in grafted tomatoes is primarily due to the overall synergistic effects of the grafting combinations [40,41]. However, the changes in antioxidant enzyme activity in melon roots appear to be more directly influenced by the expression level of the *CmDUF239-1* gene. This suggests that the response of melon roots to salt stress within the grafting system may depend more on intrinsic gene regulation rather than interactions among grafting combinations. These disparities could arise from the distinct physiological characteristics of various plant species, differences in root structures, and the complexity of the gene expression regulatory networks present in grafting systems.

### 3.3. Role of CmDUF239-1 in Enhancing Salt Tolerance in Grafted Melon by Promoting the Expression of CmSOS1, CmNHX6, CmKUP3, and CmSKOR Genes to Improve Na^+^/K^+^ Homeostasis

Regulating K^+^ and Na^+^ levels, along with the expression of their respective transport genes, is a crucial mechanism through which plants enhance their salt tolerance under stress conditions [43,44]. Both DUF family genes and grafting techniques have been shown to significantly contribute to improved salt tolerance in various crops via this mechanism. In rice, for instance, lines overexpressing the *OsDUF6* gene exhibited significantly lower Na^+^ concentrations in both leaves and roots compared to wild-type plants under salt stress, while maintaining relatively stable K^+^ levels. Consequently, the Na^+^/K^+^ ratio decreased substantially, suggesting that *OsDUF6* enhances salt tolerance by reducing Na^+^ accumulation and stabilizing K^+^ content. Additionally, *OsDUF6* upregulated the Na^+^ transporter gene *OsHKT1-4*, which increased the plant’s capacity to transport Na^+^ [21]. Similarly, the *OsDUF868.12* gene regulates the expression of the Na^+^ transport genes *OsSOS1* and *OsSOS3* in response to salt stress; the transcription levels of these genes were significantly higher in overexpression lines than in wild types, thereby enhancing cellular Na^+^ efflux and mitigating salt-induced cellular damage [36]. In *Arabidopsis thaliana*, the *ZmDUF1644* gene was shown to significantly decrease Na^+^ concentrations in the roots and stems while simultaneously increasing K^+^ levels, resulting in a lower Na^+^/K^+^ ratio. This effect was accompanied by the upregulation of the *AtSOS2* gene, further contributing to enhanced salt tolerance [23]. Grafting techniques have also demonstrated substantial efficacy in improving salt tolerance among vegetables. For example, grafted melon using pumpkin rootstocks showed a marked reduction in Na^+^ content within leaves under salt stress conditions while significantly increasing K^+^ and Ca^2+^ concentrations, thus maintaining ionic balance [45]. In grafted cucumbers, those with pumpkin roots overexpressing the CmoNAC1 gene experienced an increase in K^+^ levels in both leaves and roots alongside a decrease in Na^+^ content, resulting in a notably enhanced K^+^/Na^+^ ratio. The expression levels of the sodium transporter gene *CmoHKT1;1* and the potassium transporter gene *CmoAKT1;2* were significantly upregulated, indicating that *CmoNAC1* plays a pivotal role in modulating Na^+^ and K^+^ homeostasis by regulating sodium and potassium transporter gene expression, thereby enhancing salt tolerance. Furthermore, when the *CmoDREB2A* gene was overexpressed in pumpkin roots of grafted cucumbers under salt stress, there was a corresponding increase in K^+^ levels in cucumber leaves and roots, a reduction in Na^+^ content, and a significant enhancement of the K^+^/Na^+^ ratio. Gene expression analysis revealed that in pumpkin roots overexpressing *CmoDREB2A*, there was a notable increase in the expression of the potassium transport gene *CmoAKT1;2* and the Na^+^ transport gene *CmoHKT1;1* [30,31]. This suggests that *CmoDREB2A* significantly influences ion balance in grafted cucumbers by regulating the expression of K^+^/Na^+^ transport genes, thereby further enhancing salt tolerance.

This study investigated the mechanisms underlying salt tolerance in grafted melon under salt stress, focusing on two key aspects: the role of *DUF* genes and the impact of grafting techniques. Our findings reveal both similarities and differences when compared to results from other crops (Figure 6a–i). From a similarity standpoint, the *DUF* gene family enhances salt tolerance by regulating Na^+^/K^+^ transport-related genes, thereby influencing ionic balance across various species. For instance, *OsDUF6* upregulates *OsHKT1;4* in rice [21], while *OsDUF868.12* modulates the expression of *OsSOS1* and *OsSOS3* [44]. *ZmDUF1644* increases *AtSOS2* expression in Arabidopsis [23]. In our study, *CmDUF239-1* was shown to regulate the expression levels of *CmSOS1*, *CmNHX6*, *CmKUP3*, and *CmSKOR* in grafted melon (Figure 7b–m). Moreover, grafting techniques have demonstrated significant effects on salt tolerance across different vegetable crops. Grafting melons and cucumbers onto pumpkin rootstocks improved root ion uptake and transport, significantly lowering Na^+^ levels and raising K^+^ concentrations in leaves and roots. Nonetheless, notable differences were present between the two. Regarding the function of *DUF* genes, the specific Na^+^/K^+^ transport-related genes regulated by these genes vary among crops. For example, *OsDUF6* primarily regulates *OsHKT1;4* [21], whereas *CmDUF239-1* influences multiple genes, including *CmSOS1*, *CmNHX6*, *CmKUP3*, and *CmSKOR* in grafted melon (Figure 7b–m). These variations may be attributed to the distinct genomic backgrounds of different species and their evolutionary adaptations to salt stress. In terms of grafting techniques, the effects and mechanisms of ion regulation differ significantly among crops. Grafting melons onto pumpkin rootstocks predominantly achieves ionic balance by reducing Na^+^ levels in leaves while increasing K^+^ and Ca^2+^ concentrations [45]. Conversely, grafting cucumbers onto pumpkin roots substantially alters the expression levels of sodium-potassium transport genes through the regulation of *CmoNAC1* and *CmoDREB2A* genes, consequently affecting ion content and ratios [30,31]. This study extensively explored the influence of *CmDUF239-1* on the expression of Na^+^/K^+^ transport-related genes—*CmSOS1*, *CmNHX6*, *CmKUP3*, and *CmSKOR*—in the root systems of grafted melon (Figure 7b–m). These genes exhibit distinct regulatory patterns under salt stress: *CmSOS1* primarily facilitates Na^+^ excretion, *CmNHX6* is involved in Na^+^ compartmentalization, *CmKUP3* is responsible for K^+^ absorption, and *CmSKOR* transports K^+^ to aerial parts of the plant. By regulating these genes, *CmDUF239-1* significantly influences the contents and ratios of Na^+^ and K^+^, thus maintaining Na^+^/K^+^ homeostasis. This finding underscores the critical role of *CmDUF239-1* in enhancing salt tolerance in grafted melon and highlights the unique function of the *DUF* gene family in regulating Na^+^/K^+^ transport-related genes across various crops. This uniqueness may arise from the specialized mechanisms that various crops have developed over time to adapt to the challenges posed by salt stress.

## 4. Materials and Methods

### 4.1. Cultivation and Salt Treatment of Melon Seedlings

The melon varieties used in this experiment, ‘ST2’ (‘Shoutian No. 2’), ‘LBS9’ (‘Lvbaoshi No. 9’), and ‘XZM17’ (‘Xizhoumi No. 17’), were all obtained from Hebei Liertian Seed Industry Co., Ltd., Hebei, China. The purebred melon germplasm ‘BCG’ was supplied by the Facility Horticulture Research Group at Huazhong Agricultural University [46]. The melon seeds were initially disinfected by immersion in pure water at 55 °C for 15 min, followed by soaking at 25 °C for 6 h. Afterward, the seeds were placed in a Petri dish (10 cm in diameter) lined with three layers of moist filter paper and incubated in a constant temperature chamber at 28 °C, with a light of 60% to promote germination. Once germination occurred, the seedlings were sown into sponge blocks soaked in 1/2 Hoagland nutrient solution. When the cotyledons had fully expanded, the melon seedlings were transplanted into hydroponic cups (90 mm mouth diameter, 57 mm bottom diameter, and 135 mm height), each containing 400 mL of modified 1/2 Hoagland nutrient solution. The nutrient solution was formulated with 1 mM MgSO_4_·7H_2_O, 4 mM CaCl_2_, 60 mM KNO_3_, 0.5 mM Ca(H_2_PO_4_)_2_·H_2_O, and 74.93 mg/L of solid trace elements (DZPM0059) provided by Coolaber Company, Beijing, China. The pH of the nutrient solution was adjusted to 6.5 using 1 M KOH. Melon seedlings were cultivated under the following conditions: daytime temperature at 30 °C, nighttime temperature at 18 °C, light intensity at 250 μmol·m^−2^·s^−1^, and a light duration of 14 h. Seedlings at the two-leaf and one-bud stage were divided into treatment groups: a control group maintained with salt-free nutrient solution and a salt treatment group provided with nutrient solution containing 200 mM NaCl. [Preliminary experiments revealed that there were no significant differences in some injury indices when treated with 100 mM and 300 mM NaCl. Only under 200 mM NaCl treatment did all injury indices show significant differences, making it the appropriate salt concentration (Figure S4).] Each treatment was replicated three times, with six plants per replication. After 5 days of salt treatment, phenotypic photography was conducted, and samples were collected to assess growth indicators and damage metrics, including MDA content and REC.

### 4.2. Cultivation and Salt Treatment of Grafted Melon Seedlings

By applying the previously outlined method for self-rooted melon seedling cultivation, rootstocks from the salt-sensitive variety ‘ST2’ and the salt-tolerant variety ‘XZM17’ were produced, alongside the ‘ST2’ scion. Once the rootstock had emerged from the soil, germination of the scion was initiated. Grafting was carried out via the cutting method after the scion’s cotyledons had completely expanded [16]. Following successful grafting, the cultivation conditions were maintained for the self-rooted melon seedlings. When the grafted melon developed two leaves and one bud, they underwent salt treatment, following the same protocol used for the self-rooted seedlings. On days 1, 3, and 5 post-salt treatment, samples were randomly collected for phenotypic photography and morphological and physiological measurements. Root samples were additionally harvested and preserved at −80 °C for transcriptome sequencing analysis.

### 4.3. Determination of Chlorophyll Content and Biomass

SPAD values for the first true leaves of self-rooted and grafted melon seedlings were measured after a 5-day treatment with 200 mM NaCl, using a SPAD-502 chlorophyll meter (Konica Minolta, Tokyo, Japan). Fresh weights of the above-ground parts and root systems were measured separately to assess biomass. The samples were then dried at 105 °C for 15 min and subsequently at 72 °C for 72 h, after which their dry weights were measured [16].

### 4.4. Determination of MDA Content and Relative Electrical Conductivity (REC)

Following a 5-day treatment with 200 mM NaCl, the MDA content and relative electrical conductivity (REC) in the leaves and roots of both self-rooted and grafted melon seedlings were assessed according to the methodology described by Niu et al. [47]. For MDA determination, 0.1 g of fresh leaf or root tissue was weighed and homogenized in 5 mL of 5% trichloroacetic acid. The mixture was ground and then centrifuged at 10,000 rpm for 20 min. An aliquot of 2 mL of the supernatant was combined with 2 mL of a 0.67% thiobarbituric acid solution and placed in a water bath at 100 °C for 15 min. Absorbance was recorded at 450, 532, and 600 nm, with a solution containing all reagents but no sample serving as the blank control. The MDA content was calculated using the following formula: C (nmol/g) = (6.45 (A532 − A600) − 0.56 A450) × 5/0.1. To determine REC, 0.1 g of leaf or root tissue was rinsed thrice with distilled water, blotted to remove surface moisture, transferred to a 15 mL centrifuge tube containing 5 mL of pure water, and vacuum-treated at 0.8 MPa for 20 min. The conductivity of the extract (R1) was measured using a conductivity meter (DDSJ-308F, Leici, Shanghai, China). The samples were then boiled in a water bath for 30 min, cooled to room temperature, shaken thoroughly, and the conductivity (R2) of the extract was measured. The relative electrical conductivity was calculated using the formula: Relative electrical conductivity = R1/R2 × 100% [47].

### 4.5. Transcriptome Analysis and Gene Expression Heatmap Analysis

Transcriptome sequencing was conducted on the root systems of self-rooted and grafted melon seedlings subjected to a 200 mM NaCl treatment for 1, 3, and 5 days. The RNA sequencing was performed by Beijing Tsingke Biotechnology Co., Ltd. (Beijing, China) For reference, genome data were sourced from the Cucurbitaceae Genome Database (Cucurbit Genomics Database: http://cucurbitgenomics.org/ (accessed on 2 November 2024)), utilizing the Melon DHL92 genome v4. Rigorous quality control revealed high sequencing quality across all samples, as evidenced by Q20 values above 85% and Q30 values above 80%. Gene expression levels were quantified in terms of transcripts per million (TPM). Differentially expressed genes were identified using DESeq2 (1.32.0) software [48], with the following screening criteria: Log2 (Fold Change) > 1.0 and *p* < 0.05 [49]. The RNA-seq data have been uploaded to the NCBI under accession number PRJNA1266602. The data from the heat map analysis of gene expression levels are derived from the differentially expressed genes analyzed earlier (Log2 (Fold Change) > 1.0 and *p* < 0.05).

### 4.6. qRT-PCR Analysis and Subcellular Localization Analysis

Following the methodology outlined by Huang et al. [50], a sample weighing 0.2 g was ground in liquid nitrogen, and RNA was extracted using the TransGen TransZol kit (TransGen Biotech Co., Ltd., Beijing, China). The extracted RNA was then reverse-transcribed into complementary DNA (cDNA) with the RNAsimple kit (Tiangen, Beijing, China) and subsequently diluted to a concentration of 200 ng/μL using distilled deionized water (ddH_2_O). Quantitative reverse transcription PCR (qRT-PCR) was performed using the FC-96G instrument (Bigfish, Hangzhou, China). Each reaction consisted of a total volume of 10 μL, which included 1 μL of cDNA, 0.5 μL each of forward and reverse primers, 5 μL of 2×SYBR Green qPCR Mix (Biosharp, Hefei, China), and 3 μL of ddH_2_O. The PCR amplification protocol involved an initial denaturation step at 94 °C for 30 s, followed by 40 cycles. Each cycle included denaturation at 94 °C for 5 s, annealing at 56 °C for 30 s, and extension at 72 °C for 10 s. Gene expression levels were calculated using the 2^−ΔΔCt^ method [51], with *CmActin* serving as the internal reference gene. The sequences of the quantitative primers are provided in Table S1. The subcellular localization of the CmDUF239-1 protein was performed following the method described by Peng et al. [16].

### 4.7. Creation of Grafted Melon Materials with Root Knockout and Overexpression of CmDUF239-1

‘XZM17’ served as the rootstock and ‘ST2’ as the scion for the generation of grafted melon plants, which included root systems carrying empty vectors, *CmDUF239-1* knockout constructs, and *CmDUF239-1* overexpression constructs. The overexpression vector for *CmDUF239-1* (*MELO3C022991*) and a CRISPR/Cas9 vector were constructed based on the methodology described by Geng et al. [52], followed by transformation into Escherichia coli 5α. Subsequently, Agrobacterium K599 was transformed with the empty pKSE403 plasmid, the pKSE403 plasmid containing *CmDUF239-1* sgRNA, and the pKSE403 plasmid containing *CmDUF239-1* coding sequence (CDS), according to the K599 instruction manual (Weidi, Shanghai, China). Melon plants were infected with transformed Agrobacterium K599, and transplantation was performed four days after infection. Upon establishment, seedlings were maintained by removing non-fluorescent roots every five days and retaining red fluorescent roots. Grafting was performed when the seedlings developed one leaf and one heart. For three days following grafting, the plants were kept in the dark at 100% humidity, after which they were gradually acclimatized to light and ventilation. Before subjecting the plants to salt treatment, Hi-TOM sequencing was conducted on the red fluorescent roots of the knockout plants to assess gene editing efficiency. Additionally, real-time quantitative PCR (qRT-PCR) analysis was carried out on the red fluorescent roots of the overexpressed plants to confirm the successful overexpression of *CmDUF239-1*. Grafted melon seedlings with CmDUF239-1 knockout or overexpression in the root system were obtained following these evaluations and transplanted into hydroponic cups. When seedlings reached the two-leaf and one-heart stage, a salt-free nutrient solution was applied as the control, while 200 mM NaCl-supplemented nutrient solution was used for the salt treatment. Each treatment was replicated three times, with six plants per repetition. On the 1st, 3rd, and 5th days following salt treatment, random samples were taken to assess morphological and physiological indicators, while root samples were stored at −80 °C for further analysis. The vector construction and Hi-TOM sequencing primers are shown in Table S2.

### 4.8. Determination of SOD, POD, and CAT Enzyme Activities

The activities of SOD, POD, and CAT enzymes in the root systems of grafted melon seedlings with either knockout or overexpression of *CmDUF239-1* were assessed after treatment with 200 mM NaCl for 1, 3, and 5 days. The enzyme activities for SOD, POD, and CAT were measured using kits provided by Beijing Solarbio Technology Co., Ltd. (Beijing, China), with the following item numbers: BC5165 for SOD, BC0090 for POD, and BC0200 for CAT [53].

### 4.9. Analysis of Na^+^ and K^+^ Contents

The concentrations of sodium ions (Na^+^) and potassium ions (K^+^) in the roots of grafted melon seedlings with either knockout or overexpression of *CmDUF239-1* were analyzed after treatment with 200 mM NaCl for 1, 3, and 5 days. The procedure for determining ion content is as follows: A 0.1 g sample of leaves or roots from plants previously used for dry weight determination was transferred to a digestion tube. Five milliliters of concentrated sulfuric acid was added, and digestion was performed at 300 °C until a uniform brownish-black liquid was obtained. The solution was then treated with 30% H_2_O_2_ until clear and transparent, followed by dilution to 50 mL with ddH_2_O. The resulting solution was further diluted as necessary and analyzed using an atomic absorption spectrophotometer (Varian Spectra AA220, Cal-L Enterprises, Chatsworth, CA, USA) according to Geng et al. [52].

### 4.10. Data Statistical Analysis and Image Processing

The significance of differences in the data was evaluated using a two-factor analysis of variance (ANOVA) followed by Duncan’s new complex range test (*p* < 0.05). Statistical analysis was performed using the ‘dplyr’, ‘agricolae’, ‘tibble’, and ‘tidyr’ packages in RStudio version 4.03. Additionally, the ‘ggplot2’, ‘RColorBrewer’, ‘officer’, and ‘rvg’ packages were employed for the creation of statistical graphs.

## 5. Conclusions

**Figure 8:**
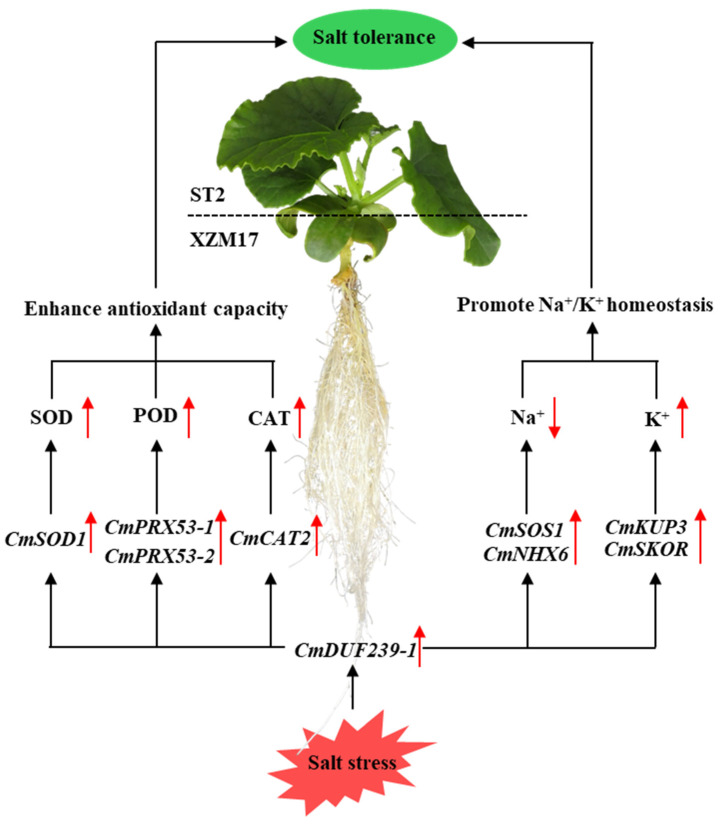
Mechanism by *CmDUF239-1* enhances salt tolerance in grafted melon. *CmDUF239-1* enhances the antioxidant capacity of grafted melon by upregulating the expression of antioxidant enzyme-related genes (*CmSOD1*, *CmPRX53-1*, *CmPRX53-2*, and *CmCAT2*) in the roots, thereby increasing the activities of SOD, POD, and CAT. Additionally, *CmDUF239-1* maintains Na^+^/K^+^ homeostasis in grafted melon by upregulating the expression of Na^+^/K^+^ transport-related genes (*CmSOS1*, *CmNHX6*, *CmKUP3*, and *CmSKOR*) in the roots.

In conclusion, the *CmDUF239-1* gene within the rootstock root system functions as a critical regulator of salt tolerance in grafted melon. This gene operates through two primary pathways: Firstly, it upregulates the expression of the *CmSOD1*, *CmPRX53-1*, *CmPRX53-2*, and *CmCAT2* genes in the root system, leading to an increase in the activities of SOD, POD, and CAT. This enhancement significantly boosts the antioxidant capacity of the plant. Secondly, *CmDUF239-1* promotes the upregulation of *CmSOS1* and *CmNHX6* gene expression, facilitating the excretion and compartmentalization of Na^+^ ions. Additionally, it enhances the expression of *CmKUP3* and *CmSKOR* genes, which improves K^+^ absorption and transport, thereby maintaining Na^+^/K^+^ homeostasis (Figure 8). Through these mechanisms, the *CmDUF239-1* gene plays a pivotal role in significantly improving the salt tolerance of grafted melon.

## Data Availability

The datasets used and/or analyzed during the current study are available from the corresponding author upon reasonable request.

## Supplementary Materials

The following supporting information can be downloaded at: https://www.mdpi.com/article/10.3390/plants14172670/s1. Figure S1: Evaluation of salt tolerance in four melon varieties. After a 5-day treatment with 200 mM NaCl, the phenotypes of the four melon varieties are shown (a), along with their dry weight of shoot (b), dry weight of root (c), leaf malondialdehyde (MDA) content (d), root malondialdehyde (MDA) content (e), leaf relative electrical conductivity (REC) (f), and root relative electrical conductivity (REC) (g). Mean ± SE (n = 3). Different lowercase letters indicate significant differences among treatments (*p* < 0.05). Figure S2: Principal component analysis (PCA) and differential gene count analysis of root transcriptome data from self-grafted and grafted melon under salt stress. PCA of root transcriptome data from self-grafted and grafted melons treated with 200 mM NaCl for 1 day (a), 3 days (b), and 5 days (c). (d) Analysis of differential gene counts in the roots of self-grafted and grafted melons after 1, 3, and 5 days of 200 mM NaCl treatment. ST2/ST2 : Self-grafted melons using ‘ST2’ as both the rootstock and scion, ST2/XZM17: Grafted melons with ‘XZM17’ as the rootstock and ‘ST2’ as the scion. Figure S3: Quantitative analysis of *CmDUF239-1* gene expression under salt stress and subcellular localization analysis of *CmDUF239-1*. Real-time quantitative PCR (qPCR) analysis of *CmDUF239-1* gene expression in the roots of self-grafted and grafted melons treated with 200 mM NaCl for 1 day (a), 3 days (b), and 5 days (c). (d) The expression level of the *CmDUF239-1* gene in different tissues of ‘XZM17’ under salt stress for 1 day. (e) Subcellular localization analysis of *CmDUF239-1*. Different lowercase letters indicate significant differences among treatments (*p* < 0.05). Mean ± SE (n = 3). Different lowercase letters indicate significant differences among treatments (*p* < 0.05). ST2/ST2 : Self-grafted melons using ‘ST2’ as both the rootstock and scion, ST2/XZM17: Grafted melons with ‘XZM17’ as the rootstock and ‘ST2’ as the scion. Figure S4: Screening of optimal salt concentration for melon salt treatment. Effects of different salt concentrations on the phenotypes (a), chlorophyll content (b), fresh weight of shoots (c) and roots (d), dry weight of shoots (e) and roots (f), relative electrical conductivity of leaves (g) and roots (h), and malondialdehyde content in leaves (i) and roots (j) of two melon cultivars. Table S1: Primers of q-RT PCR. Table S2: Primers for Hi-TOM sequencing and vector construction.

## Abbreviations

The following abbreviations are used in this manuscript:

SOD	superoxide dismutase
CAT	Catalase
DUF	Domain of Unknown Function
POD	Peroxidase
PAL	phenylalanine ammonia-lyase
MDA	malondialdehyde
DEGs	differentially expressed genes
PCA	principal component analysis
REC	relative electrical conductivity
WT	wild-type
ROS	reactive oxygen species
SG	self-grafted
cDNA	complementary DNA
qRT-PCR	real-time quantitative PCR
H_2_O_2_	hydrogen peroxide
