# Supplementary material for: CmDUF239-1 Improves the Salt Tolerance of Grafted Melon by Enhancing Antioxidant Capacity and Na+/K+ Homeostasis
Source: Plants (Basel). 2025 Aug 27;14(17):2670. doi: 10.3390/plants14172670 (PMC12430171; doi:10.3390/plants14172670)
Supplement: Supplementary file 1 [file plants-14-02670-s001.zip › plants-3773944-supplementary.docx]

**
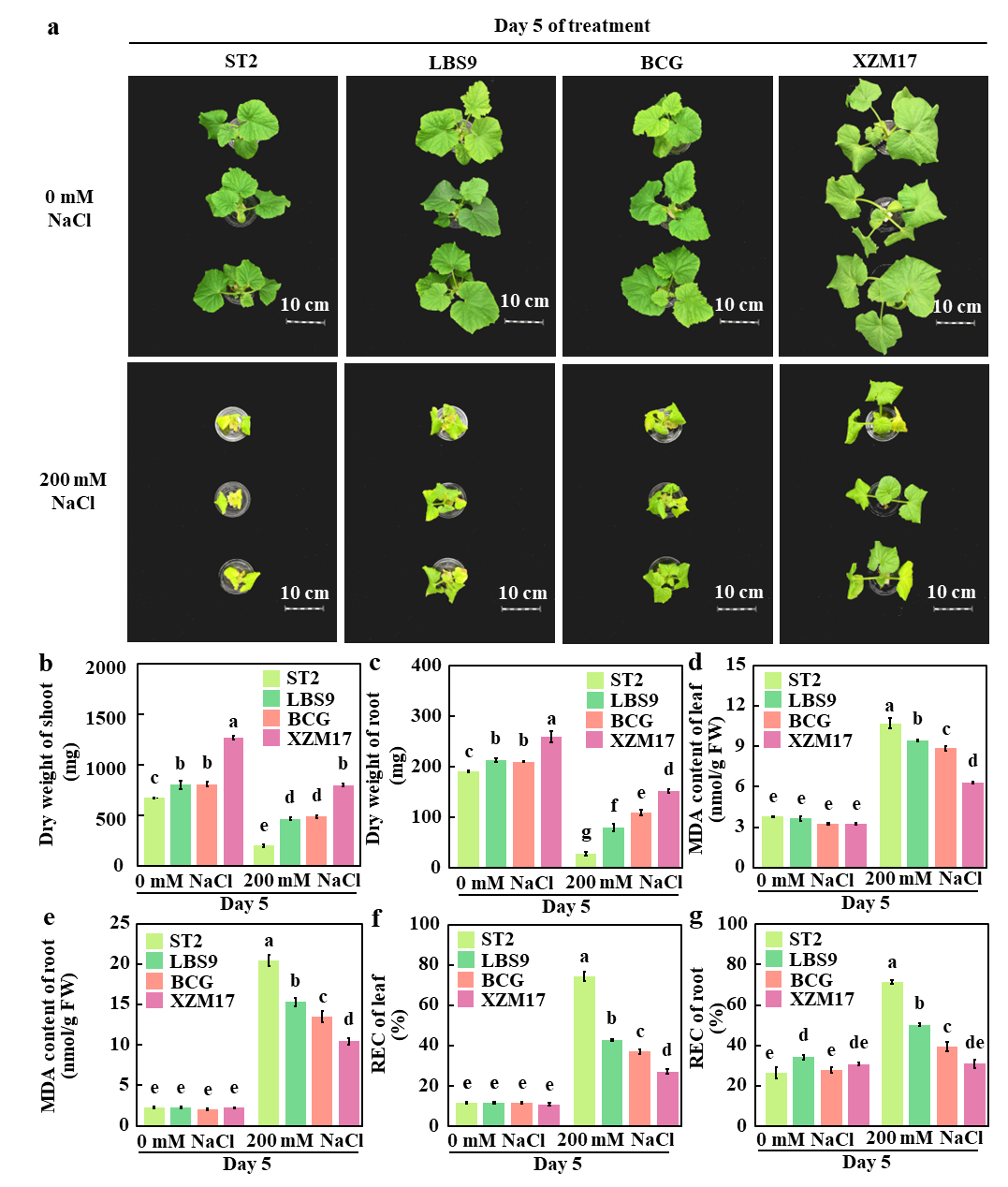
**

**Figure S1 Evaluation of salt tolerance in four melon varieties.** After a 5-day treatment with 200 mM NaCl, the phenotypes of the four melon varieties are shown (a), along with their dry weight of shoot (b), dry weight of root (c), leaf malondialdehyde (MDA) content (d), root malondialdehyde (MDA) content (e), leaf relative electrical conductivity (REC) (f), and root relative electrical conductivity (REC) (g). Mean ± SE (n = 3). Different lowercase letters indicate significant differences among treatments (P < 0.05).

**
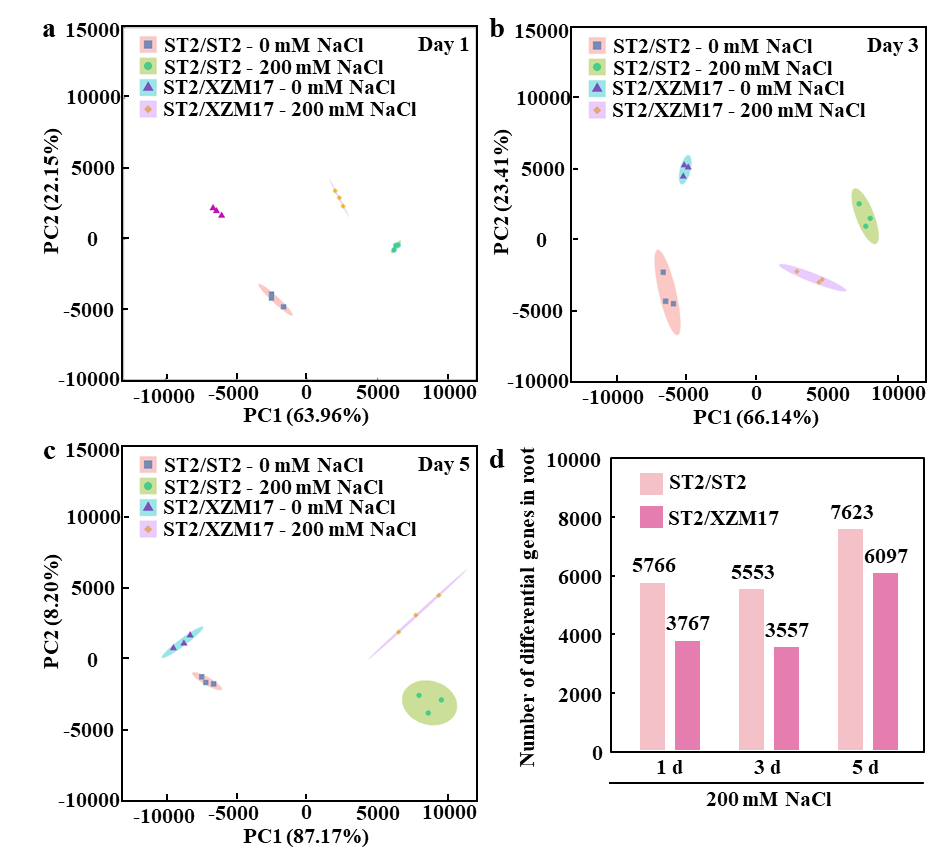
**

**Figure S2 Principal component analysis (PCA) and differential gene count analysis of root transcriptome data from self-grafted and grafted melons under salt stress.** PCA of root transcriptome data from self-grafted and grafted melons treated with 200 mM NaCl for 1 day (a), 3 days (b), and 5 days (c). (d) Analysis of differential gene counts in the roots of self-grafted and grafted melons after 1, 3, and 5 days of 200 mM NaCl treatment. ST2/ST2 : Self-grafted melons using ‘ST2’ as both the rootstock and scion, ST2/XZM17: Grafted melons with ‘XZM17’ as the rootstock and ‘ST2’ as the scion.

**
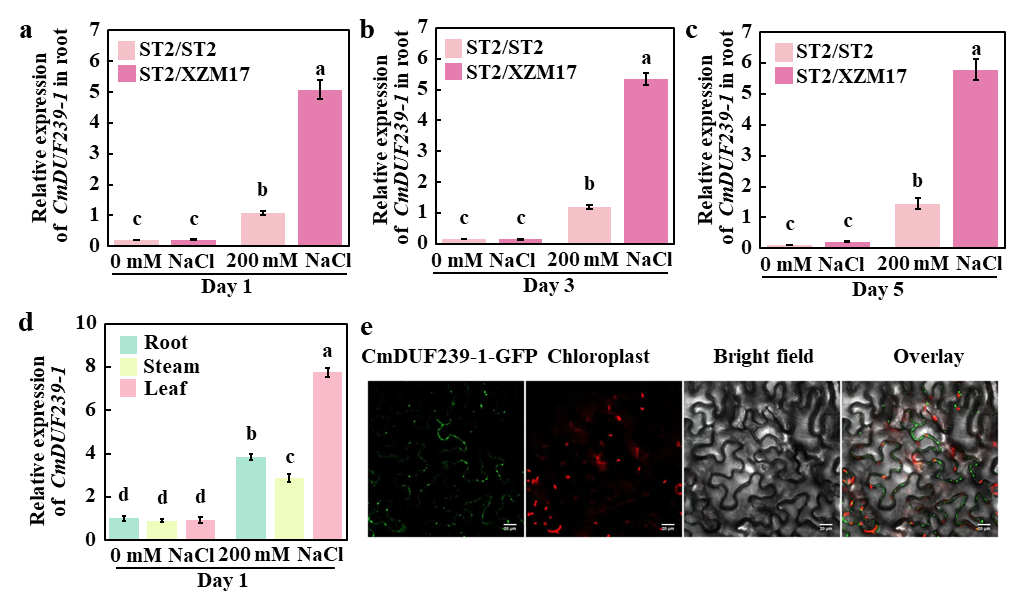
**

**Figure S3 Quantitative analysis of *CmDUF239-1* gene expression under salt stress and subcellular localization analysis of *CmDUF239-1.*** Real-time quantitative PCR (qPCR) analysis of *CmDUF239-1* gene expression in the roots of self-grafted and grafted melons treated with 200 mM NaCl for 1 day (a), 3 days (b), and 5 days (c). (d) The expression level of the *CmDUF239-1* gene in different tissues of ‘XZM17’ under salt stress for 1 day. (e) Subcellular localization analysis of *CmDUF239-1*. Different lowercase letters indicate significant differences among treatments (*P* < 0.05). Mean ± SE (n = 3). Different lowercase letters indicate significant differences among treatments (*P* < 0.05). ST2/ST2 : Self-grafted melons using ‘ST2’ as both the rootstock and scion, ST2/XZM17: Grafted melons with ‘XZM17’ as the rootstock and ‘ST2’ as the scion.

**
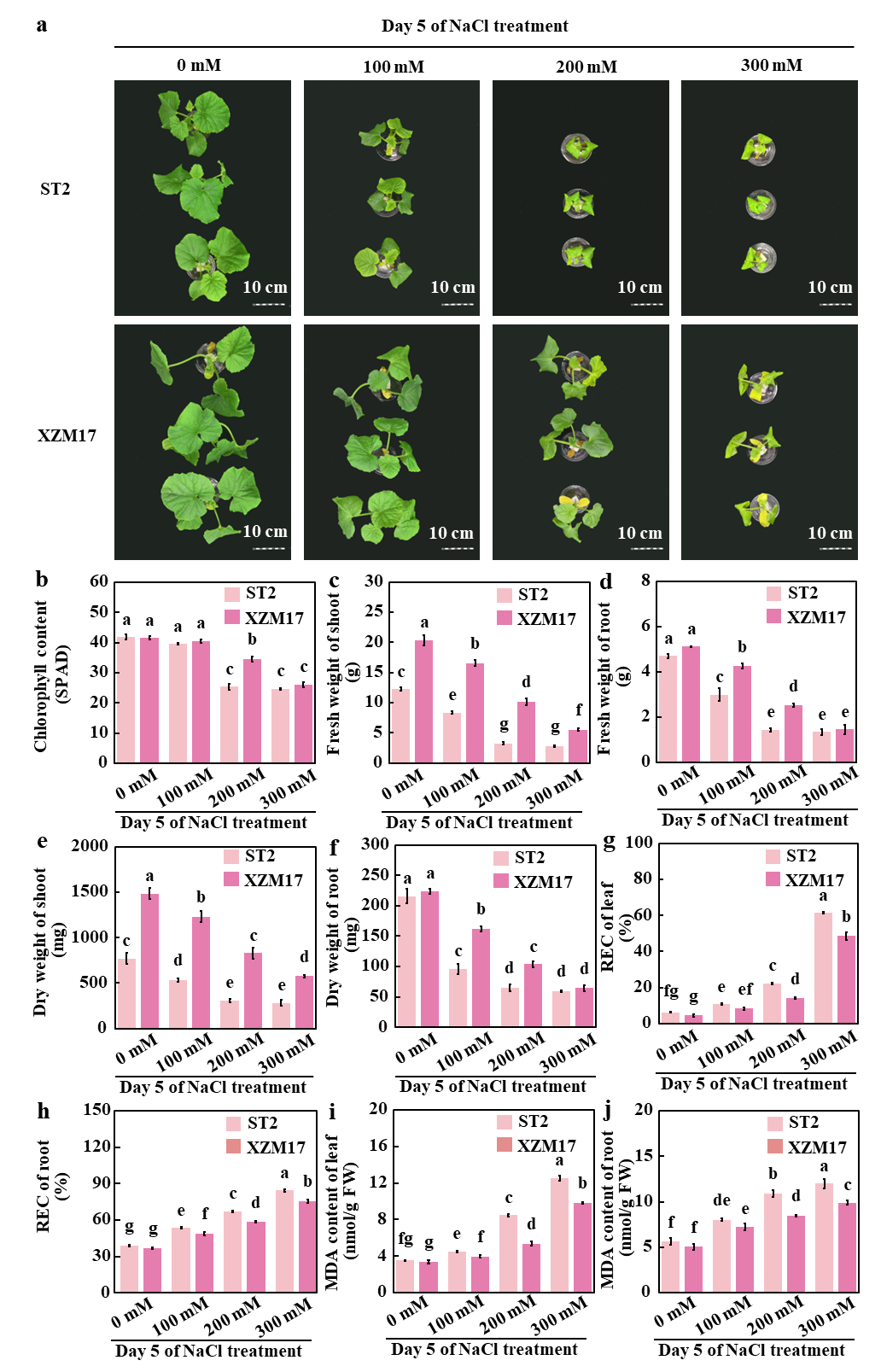
**

**Figure S4. Screening of optimal salt concentration for melon salt treatment.** Effects of different salt concentrations on the phenotypes (a), chlorophyll content (b), fresh weight of shoots (c) and roots (d), dry weight of shoots (e) and roots (f), relative electrical conductivity of leaves (g) and roots (h), and malondialdehyde content in leaves (i) and roots (j) of two melon cultivars.

**Table S1 Primers of q-RT PCR**

| **Gene name** | **Gene ID** | **Forward Primer** | **Reversed Primer** |
| --- | --- | --- | --- |
| ***CmActin*** | ***MELO3C025848*** | **TGGTCGTATTGAAATGGGTC** | **CTCGTCAGCGAACTTCTCTC** |
| ***CmDUF239-1*** | ***MELO3C022991*** | **TGGCAATCAGTTGGAAGAAG** | **AACCACCACAGACTCACTCC** |
| ***CmSOD1*** | ***MELO3C015374*** | **TGCTCCCGAGGACGAGAA** | **CACAACCGCCCTTCCGAT** |
| ***CmPRX53-1*** | ***MELO3C014653*** | **ACGCCAACTTTGTGCCAC** | **AGCCCCAGGGGTTGAGAA** |
| ***CmPRX53-2*** | ***MELO3C014656*** | **CGCCAACGCTGCCATTAC** | **GACAACGTTGGCCAAGCG** |
| ***CmCAT2*** | ***MELO3C017023*** | **TGCACTGGGAAGCGTGAG** | **CCGTTCCTGCCTGTCTGG** |
| ***CmSOS1*** | ***MELO3C005228*** | **TGGGCGCCTTTGGAGATC** | **TTGTGAGGGTGCAGTGGC** |
| ***CmNHX6*** | ***MELO3C015375*** | **GGGGTCGGCACTGAGAAC** | **CCTTGTCCGCTGCTCGAA** |
| ***CmKUP3*** | ***MELO3C014763*** | **GCAGCAAATCCGCCACAC** | **ACCTCACTTGGCGCCTTC** |
| ***CmSKOR*** | ***MELO3C022820*** | **GTGAGCGGCGGAAGAGAG** | **TCACGAACGCTCTCCACC** |

**Table S2 Primers for Hi-TOM sequencing and vector construction**

| **ID** | **Forword primer** | **Reverse primer** |
| --- | --- | --- |
| **403R-*CmDUF239-1*** | **ctcgagtaatctagaATGGGGGTGCTGATTTATATTA** | **gaaagctctgagctcTTACTTGCCGGAGAACCACCAC** |
| **sgRNA-*CmDUF239-1*** | **attgAAAAGTTGCAGTGATTTGA** | **aaacTCAAATCACTGCAACTTTT** |
| **HITOM-sg*CmDUF239-1*** | **gagtacggtgtgcATGGGGGTGCTGATTTATATTA** | **ggatgctggatggTTACTTGCCGGAGAACCACCAC** |
